# Home-Based Physical Activity as a Healthy Aging Booster before and during COVID-19 Outbreak

**DOI:** 10.3390/ijerph19074317

**Published:** 2022-04-04

**Authors:** Marianna De Maio, Cecilia Bratta, Alice Iannaccone, Loriana Castellani, Carl Foster, Cristina Cortis, Andrea Fusco

**Affiliations:** 1 Department of Human Sciences, Society and Health, University of Cassino and Lazio Meridionale, Viale dell’Università, 03043 Cassino, Italy; marianna.demaio@unicas.it (M.D.M.); cecilia.bratta@unicas.it (C.B.); alice.iannaccone@unicas.it (A.I.); l.castellani@unicas.it (L.C.); andrea.fusco@unicas.it (A.F.); 2 Department of Exercise and Sport Science, University of Wisconsin-La Crosse, La Crosse, WI 54601, USA; cfosteruwl@gmail.com

**Keywords:** exercise, older adults, SARS-CoV-2, well-being

## Abstract

The role of physical activity in improving overall aspects of health regardless of age is well documented. Due to the coronavirus disease 2019 outbreak, preventive measures to limit airborne infection have been introduced, with people, especially older adults, advised to stay at home, thus increasing sedentary lifestyle and the risk of chronic diseases. As one of the few possible ways to stay active is home-based training, this review aims to provide evidence on alternative and feasible home-based activity programs as a tool to improve the fitness level in older adults, especially when preventive measures are needed to ensure isolation and limit interpersonal contacts. During quarantine, older adults, especially those with chronic diseases, are recommended to regularly exercise. Combined balance and muscle-strengthening training has proven to be particularly useful in limiting falls and mobility limitations. In addition, the use of virtual reality systems seems to be a potential strategy in remaining physically active, reducing physical inactivity time and significantly increasing the compliance of the older adults with physical activity programs. In conclusion, home-based programs induce improvements in physical functions in general and quality of life in older people with or without co-morbidities, and it can be considered in the future as one of the feasible and economic ways to increase physical well-being. This may be of unique importance in the setting of coronavirus disease 2019 enforced limitations in out-of-home activity.

## 1. Introduction

The beneficial effects of physical activity (PA) in improving overall aspects of health regardless of age are well documented [1]. In fact, PA may contribute to the maintenance of general well-being [2], counteracting the detrimental effects of aging, reducing the risk of falls, improving postural control and quality of life (QoL) in general [3]. PA is a protective factor for the management and prevention of noncommunicable diseases such as sarcopenia, type 2 diabetes, fractures, osteoarticular disorders and pulmonary disease [4]. Despite its beneficial effects, insufficient PA has been associated with 1.5–3% of direct health care costs [5] and the World Health Organization (WHO) considers insufficient PA as the fourth leading risk factor for global mortality. Thus, to increase the amount of PA in the general population, especially in apparently healthy older adults or those with specific chronic diseases, the WHO published new 2020 guidelines on PA and sedentary behavior [4] recommending to engage in 150–300 min per week of moderate-intensity aerobic PA or 75–150 min per week of vigorous-intensity aerobic PA on at least two days per week. Older adults should also perform functional balance and strength training at moderate or greater intensity at least 3 days per week.

With the spreading of the coronavirus disease 2019 (COVID-19) pandemic, health authorities adopted preventive measures, such as limiting participation in several activities, including sport, exercise and PA outside the home. Those limitations have led to an increase in sedentary behavior and reduced levels of PA (65 min per week), which has increased the risk of chronic health conditions in older adults [6]. In this context, PA carried out in the home setting is recommended as an effective measure to stay healthy [7,8,9], boost immune system functioning [10,11], improve general physical functioning [12] and physical fitness [13]. Home-based PA programs represent a potentially effective strategy for reducing sedentary behaviors, social isolation/distancing health consequences and promoting enjoyable forms of PA during the COVID-19 pandemic. In fact, during quarantine, there has been a proliferation of supervised or unsupervised online tutorials and classes for exercising at home, which are easily accessible with tablets, iPads, personal computers and video consoles [14], all designed to promote PA. While the use of technological devices has usually been associated with sedentary lifestyles and a decline in PA [15], to date it has been demonstrated that home-based PA activity supported by technology [3,16] has induced improvements in postural control and cognitive capabilities, a reduction in risk of falls and contributed to maintaining the regular practice of PA in a safe home environment.

Moreover, it has been demonstrated how sedentary behaviors, especially during the COVID-19 quarantine, may lead to adverse changes in older individuals’ lifestyle, with several consequences such as reduction in endurance capacity, loss of muscle strength, becoming overweight and the onset of chronic conditions [17,18]. In this context, there are several well-known beneficial effects of regular PA that contribute to improving health in older adults [19]. The purpose of this narrative review is to investigate the literature dealing with the effects of home-based PA for older adults (>65 years old [20]), specifically focusing on postural control, fall prevention, mobility, strength and QoL, before and during the COVID-19 pandemic. Concerning this purpose, a narrative review has been carried out to help understand the beneficial effects of active lifestyles, particularly those related to older individuals before and during the COVID-19 pandemic. This narrative review can be a valuable source for health professionals and stakeholders interested in improving health lifestyles and reducing sedentary behaviors. In addition, this narrative review could inspire further research to better understand the role of PA before and during restriction situations such as the COVID-19 outbreak.

The paper is organized in main paragraphs focusing on the beneficial effects of home-based balance programs in preventing falls, the effectiveness of home-based PA programs in reducing mobility limitations, the beneficial effects of home-based strength-resistance training programs and the beneficial role of PA carried out at home on chronic diseases, before and during the COVID-19 pandemic. In addition, detailed information about frequency (how often), intensity (how hard), time (duration or how long), type (mode or what kind), volume (amount) and progression (advancement) or the FITT-VP (frequency, intensity, time, type, volume and progression) principle of exercise prescription [21] of each home-based PA programs are provided.

### Literature Search

We conducted a computer search for English language literature from the databases of Scopus, Web of Science and PubMed. We included studies that assessed home-based PA programs before COVID-19 and during the COVID-19 pandemic outbreak (from January 2004 to November 2021) among the general population of individuals older than 65 years old (mean age: 68.2 ± 7.2 years old). Keywords used included ‘home-based physical activity programs‘, ‘COVID-19’, ‘quality of life’, ‘balance and falls’, ‘mobility’, ‘strength-resistance training’, ‘chronic diseases’, ‘technological device’ and ‘older adults’. The data extracted have been evaluated and reviewed by the authors.

Initially, 513 studies were identified in the databases. After eliminating duplicates and reading articles’ titles and abstracts, there were 124 studies. Lastly, according to inclusion criteria, a total of 57 articles were reviewed.

## 2. Home-Based Programs before COVID-19

For apparently healthy older individuals [21], PA is commonly represented by leisure time (sports or planned) exercises, transportation (walking or cycling) and/or daily household chores in home or community settings [4]. On the other hand, more and more older adults spend most of their awake time in sedentary activities [22]. To cope with these conditions and to fulfill regular levels of PA, adapted programs carried out at home are widely used [23]. Home-based programs can counteract sedentary behaviors, promote subjects’ integration, support social relations, and improve overall health-related physical parameters among frail populations. Moreover, to improve older adults’ physical capacities, such as balance, mobility or strength, several adapted home-based PA programs have been used.

### 2.1. Balance and Falls

Maintaining postural control is an important ability of human movement, necessary in all basic aspects of daily life, such as activities like walking, sitting and/or getting up and in preventing falls, especially in older adults [24]. However, studies show that the incidence of falls is three times higher among older adults with respect to young adults, increasing the 1-year mortality among fallers [25]. In particular, falls have a negative impact on the QoL of individuals because they may involve temporary limitation of mobility or disability with a consequent loss of independence [26]. To reduce the incidence of falls in older adults, health professionals and researchers have developed several home-based PA programs to aid older adults at greater risk of falling. Therefore, the development and implementation of effective strategies to improve balance and prevent falls is fundamental [27].

*Balance training and falls using technological devices.* Home-based postural control programs using technological devices may reduce the risk of falls in older people. A long-term (24 months) balance training program with a frequency of 1–3 times per week using tablets, was associated with a balance improvement and an increase in confidence [3]. In addition, home-based short-term (8 weeks; 3 times per week) programs using iPads, with individualized exercises, were useful for the prevention of cognitive decline and the integration of functional physical abilities [16]. Evidence [3,16] underlined the importance of home-based PA programs using technological devices, improving the execution of regular PA levels and normal activities of daily life, such as walking and/or getting up, thus favoring autonomy and independence in older adults.

*Balance training and falls in community dwellings older people.* Among community-dwelling older adults, PA interventions have been shown to contribute to improved PA levels and functional outcomes [16]. Walking training programs lasting from 8 [28] to 16 weeks [29] proved to be effective in preventing falls. Supervised low-intensity walking programs (3 times per week) improved strength, proprioception, balance, flexibility, and functional mobility, leading to a reduction in the risk of falls [28,29]. Evidence [4] suggests that PA programs focused on balance, strength and gait or a combined program are associated with a reduced risk of falls, bone fragility and the risk of fall injuries in the elderly. It has been also demonstrated that multicomponent (i.e., well-rounded program that combine endurance, strength, coordination, balance and flexibility exercises) programs especially focusing on balance and strength training, in 3 times per week 60-min sessions, prevented bone fragility, improved physical fitness and QoL both in home-dwelling elderly women (initial physical capacity: 6.1 ± 1.2 s (timed up and go test)) [30,31] and men (initial physical capacity: 56 points on Berg balance scale) [32]. Moreover, Hatha Yoga lessons, including breathing exercises, slow dynamic, gentle muscle and joint movements (shoulder/arm/wrist circling, neck rolling) and balance exercises such as heel/toe rising, standing on one leg at a time, walking backwards or sideways, walking heel to toe in a straight line, improved balance function, reduced risk of falls and fear of falling and thereby improved community dwelling older adults’ QoL [33]. Therefore, home-based PA programs focused on walking, in home settings, balance and strength exercises play a crucial role in improving physical function and decreasing the risk of falls or injury in general in older adults.

### 2.2. Mobility

Mobility is considered an important aspect of the aging process [34]. In fact, limitations in mobility are more and more frequent in older adults and affect 35% of people aged 70 to over 85 years [35] and are associated with an increased risk of falls and a decrease in QoL [34]. Sedentary behavior and inactivity are also related to mobility limitations [34]. Evidence [36] suggests that regular PA could prevent this type of limitation. For this reason, the development of effective strategies to prevent mobility limitation in older adults is fundamental. An individualized home-based, supervised program with moderate to hard intensity exercises (aerobic, muscle-strengthening and stretching) improved lower limb mobility in older adults with total knee arthroplasty (initial physical capacity: gait speed 1.2 m/s (6-min walking test)) [37]. Evidence also suggested that a semi-supervised home-based program (with on-site guidance through a home visit every 15 days) focused on aerobic, muscle-strengthening, coordination, balance and flexibility exercises (at a target RPE of 13–15) for 12 weeks, 3 times per week, caused improvements in functional mobility and QoL in sedentary older adults (initial physical capacity: <19 s (timed up and go test)) [38]. Turunen and colleagues [39] reported a long-term (6 months) individualized and multicomponent home-based program (balance, muscle-strengthening and low-intensity walking) promoting regular levels of PA that improved mobility in home-dwelling older adults. Thus, home-based programs, in particular multicomponent programs involving balance, muscle-strengthening and walking, seems to be a potential strategy to stay physically active and reduce mobility limitations in older adults.

### 2.3. Strength-Resistance Training

The aging process is characterized by muscle strength decline [40] which induces an increased risk of falls [41], reduced walking speed and loss of independence [42]. Evidence suggests that resistance training [43] and multicomponent training (balance and strength-resistance exercises) [44] are often used to increase muscle strength. Thus, encouraging and developing multicomponent home-based programs represents a good modality to reduce functional deterioration in muscle strength. A home-based resistance training program (12 weeks; 3 times per week; 10–15 repetitions; 3 sets for sessions) including biceps curl, triceps extension, lateral raise, seated row, bench press, abdominal crunch, calf raise, chair squat, knee extension, knee flexion, hip flexion, and dorsiflexion exercises, performed at low-intensity, walking at least 5000 steps during sessions and 10,000 steps on rest days, improved strength performance and muscle quality in free-living older adults (initial physical capacity: minimal PA level 150 min/week) [45]. In addition, a multicomponent semi-supervised (home visit each month) home-based program including balance exercises such as circle turns, plantar flexion ankle dorsiflexion, and tandem walk (with or without hand support) and strength exercises such as knee extension, standing hip extension, standing hip abduction, press, biceps curl and triceps extension (6 months; 3 times per week; 8 repetitions at a RPE of 7–8 on the 10-point Borg scale; 2 sets per sessions) enhanced physical functioning [44]. Additionally, in older women with osteoporosis, semi-supervised (home visits every 3 months) home-based muscle training (12 months) for upper and lower limbs improved QoL and muscle strength [46]. Moreover, a recent study [47] compared supervised (by an instructor) and unsupervised (at home) multicomponent programs (performing the same protocol) in healthy older adults. The protocol consisted of static (upright, bipodalic stance) and dynamic balance (walking) (12 weeks; 3 times per week; 30 s for 4 repetitions per session) and strength exercises for the lower extremities and trunk muscles: squats, plank, standing side leg lifts, calf raises/toe raises and standing trunk extensions (12 weeks; 3 times per week; 8–15 repetitions; 3 sets per session). It resulted in improvements in preventing injuries. However, the results suggest that supervised training was more effective than unsupervised. Therefore, multicomponent home-based PA programs focused on strength-resistance and balance exercises may be able to counteract muscle strength decline and promote regular PA levels in safe and comfortable home settings in older (Table 1 and Table 2).

### 2.4. Chronic Diseases

For adults living with chronic health conditions such as pulmonary disease, fractures, sarcopenia, type 2 diabetes and osteoarticular disorders, PA represents an effective strategy to cope with the negative effects of these diseases on QoL [4].

*Pulmonary diseases.* Pulmonary diseases are one of the main causes of morbidity and mortality. To reduce the related symptoms and improving QoL, pulmonary rehabilitation programs are recommended. A 6-month supervised home-based pulmonary rehabilitation program (3 times per week) involving stretching, breathing, balance (turn the hips in a circular motion, multidirectional step), muscle strengthening (lunge exercise, push-ups) and dual-task training (box step and clap hands) reduced the risk of falls and aspiration pneumonia [48]. Similarly, a pulmonary rehabilitation program with low-intensity exercises including sitting calisthenics, respiratory muscle stretching, walking in home environments for at least 15 min and inspiratory muscle exercises contributed to improving physiological factors and QoL in older adults with chronic obstructive pulmonary disease (COPD) [49]. Moreover, a 7-week PA program (two sessions (in the morning and in the afternoon) per day; 6 days per week) including inspiratory muscle training induced improvement in inspiratory pressure and the perception of well-being in patients with moderate to severe COPD [50].

*Fractures.* Fractures can be highly debilitating and negatively affect QoL and physical functioning, leading to a loss of independence [51]. Therefore, effective strategies, such as PA programs, are fundamental to prevent fall-related fractures. Supervised and unsupervised home-based exercise programs involving function-oriented exercises (such as getting up from a chair, climbing a step) [52] or balance with 1–2 sets of 8–10 repetitions (weight-bearing exercises, change in position/direction), muscle-strengthening (ankle dorsiflexors, knee extensors and hip abductors) with 2–3 sets of 10–15 repetitions from 6 to 24 months (two times per week) [53] resulted in improvement in physical functions among patients (initial physical capacity: 5.9 ± 2.8 points on short physical performance battery) following standard rehabilitation after hip fracture.

*Sarcopenia.* Sarcopenia is a progressive and generalized skeletal muscle disorder associated with an increased risk of occurrence of adverse events including falls, fractures, physical disability and mortality [54]. It is recognized as a major clinical problem for older people and as a real public health issue for society [55]. Regular exercise may extend life expectancy in aging people affected by sarcopenia. A 9-week supervised home- based exercise training program (three sessions per week) for sedentary care residents, including leg press and electric stimulation exercises [56], and a 3-month supervised home-based resistance (knee extensor-flexor, hip abductor-extension, ankle plantar flexors- dorsi flexors, wall push up, seated bicep curl, seated triceps extension, seated lateral shoulder raises, seated abdominal crunches: 12 repetitions; 2 sets) and balance (walking and turning around, walking- backward tandem-heel-toe- heel to toe-sideways, one leg stand, tandem stance: 12 repetitions; 2 sets) exercises program [57] showed significant improvements in physical condition, balance, muscular functional/physical performance, and a positive influence on QoL in older adults with sarcopenia (initial physical capacity: 8.42 ± 1.95 s (timed up and go test)).

*Type 2 diabetes.* Type 2 diabetes is a chronic metabolic disease characterized by hyperglycemia associated with impaired carbohydrates, lipids, and protein metabolism, resulting from inadequate insulin secretion or decreased sensitivity. It results in profound metabolic effects and its complications are among the leading causes of morbidity and mortality [58], especially in older individuals. Home-based PA programs may contribute to reducing risk factors such as high blood pressure, body weight and blood lipids (cholesterol) in people with type 2 diabetes [21]. In particular, aerobic activity (150–300 min at moderate-intensity or 70–150 min of vigorous-intensity per week) and muscle-strengthening (moderate or greater intensity on 2 or more days per week) improve the above-mentioned markers of disease progression in older adults with type 2 diabetes [4]. Furthermore, high-intensity resistance exercise (bench press, bicep curl, triceps extension, hip flexion, hip extension at 50–80% 1 repetition maximum) has been shown to elicit positive effects in reducing the glycosylated hemoglobin HgbA1c) and insulin levels [59]. In fact, high-intensity resistance exercises improve QoL, stimulate insulin sensitivity, increase muscle mass and facilitate glucose clearance [59]. Furthermore, a 6-month program involving aerobic and strength exercises [60] improved QoL, glycemic control and weight in type 2 diabetes older patients.

*Osteoarticular disorders.* Osteoarticular disorders are a common condition and responsible for functional limitations in older adults [21]. However, it has been demonstrated that low-intensity home-based PA programs improve exercise capacity in older persons with osteoarticular disorders [61]. A low-intensity walking program based on two daily sessions, 10-min (6 days per week) sessions including home intermittent walking sessions (1 min training and 1 min rest in sitting position) seems to be a good and safe alternative to stay physically active and improve exercise capacity in older people [61]. However, high-intensity exercises did not improve physical functioning and QoL in older individuals with osteoarticular disorders [62]. In fact, evidence [21] underlined the role of low-intensity PA (walking and/or swimming) in eliciting beneficial effects on pain, physical function, and QoL in subjects living with these types of disorders. In particular, low-intensity exercises have been shown to not stress joints [63]. Therefore, multicomponent home-based PA programs (strength-resistance, balance, and aerobic trainings) are more effective in improving physical functioning and QoL in sarcopenia, type 2 diabetes, fractures and pulmonary disease. However, presented data are not enough to understand the effects of balance, mobility, and strength-resistance home-based PA programs in subjects with osteoarticular conditions (Table 3 and Table 4).

## 3. Home-Based Programs during COVID-19

Despite the benefits of preventive measures (social/physical distancing and limitations of several activities including sport, exercise and PA) adopted during the COVID-19 outbreak to reduce the airborne virus transmission, its consequences led to a reduction in PA [64] and to an increase in sedentary behaviors, identifying it as a new risk factor for health [65]. Sedentary behaviors are commonly characterized by television viewing, computer use and sitting time [66]. Evidence showed that greater time spent in sedentary activities is related to an increased risk of physical limitations [67] and cognitive impairment [68] among older adults. Therefore, to counteract the detrimental effects of sedentary behaviors, home-based PA programs represented an attractive solution for maintaining physical activity [17] when home confinement is necessary.

### 3.1. Balance and Falls

According to the WHO recommendations [4], older adults should perform balance exercises on 3 or more days per week to reduce the rate of falls. In particular, it has been shown that in older adults performing balance and/or multicomponent programs focusing on strength-resistance and functional exercises, the falls rate can be reduced [47] as compared to sedentary behavior, which is associated with an increase in the number of falls [69]. During quarantine, balance programs have been defined as easy and feasible to perform for older adults, especially since they do not require specific equipment [13]. Trainings include several exercises, such as bipodalic or monopodalic stance, tandem or semi-tandem stance, walking on stable or unstable platforms, tandem gait and eye–hand or eye–foot coordination [70]. Difficulty can be progressively increased with different components such as visual or no visual information, arm position, type of surfaces and/or changes in direction [71]. Moreover, balance programs commonly include both fine and gross motor skill exercises [13]. In particular, a supervised home-based program focused on proprioceptive exercises (20 sessions; 2 times per week) including 5 min of gait training on a platform (forward, backward, sideways, on toes during eyes open and eyes closed) and proprioceptive handgrip exercise (25 repetitions of the grip exercise with both hands (alternating the side) using a ball) improved postural control and manual dexterity, reduced risk of falls and prevented sedentary behavior during quarantine [72]. Therefore, when social distance and confinement are required, home-based programs focusing on balance and motor skills exercises aimed at improving postural control and manual dexterity and reducing the rate of falls are commonly used in older adults. Other valuable and enjoyable forms of PA during quarantine are represented by exergames [73], which are able to encourage regular PA levels, decrease social isolation and improve postural control in older adults [74,75]. In addition, exergames have been shown to be a way to maintain physical therapy at home as part of the rehabilitation in improving static and dynamic balance in individuals with multiple sclerosis. Lastly, home-based exergames are considered to be both feasible and safe, and with a good compliance and adherence due to its motivating and enjoyable character [73].

### 3.2. Mobility

Evidence [76] suggests that sedentary behavior is negatively associated with physical function, especially mobility, in older adults. Thus, maintaining mobility in older age is a primary goal, as loss of mobility may predict the loss of independence [77]. During the quarantine, several home-based PA programs have been proposed for older populations. In a previous study [78], a semi-supervised home-based program (with guidance through phone calls or video chat once a week) performed at least 5 days per week, 20-min per day, for 7 weeks, was used. The home-based program consisted of quadriceps sets, hamstring sets, ankle pumps, terminal knee extension with weight, straight leg raises with weight in the supine and side-lying positions, cycling, and prone, hip, and knee flexion-extension with weight in supine, knee flexion-extension with weight in prone, and sitting positions, static stretching exercises for hamstrings and gastrosoleus muscles, as well as a low stool-assisted knee joint bending exercise which resulted in better range of motion in older persons with knee arthroplasty. In addition, 8 weeks of a multicomponent home-based program (muscle strength, balance and mobility) improved functional mobility among older adults [79]. Therefore, the promotion of home-based programs focusing on mobility during quarantine represented a good strategy to improve functional mobility among older adults. However, data were not conclusive to evaluate the effect of mobility home-based programs during quarantine. Moreover, guidelines to help optimize benefits while minimizing potential risks when performing home-based PA programs could be useful.

### 3.3. Strength-Resistance Training

To promote strength-resistance training and to improve compliance, home-based programs have been considered as low-cost strategies to increase PA among older adults [12]. Strength-resistance training can be easily performed in a safe home environment with minimal resources, such as jumps, dips, push-ups, and/or squats with or without support. They can also be performed in reduced space, such as a home environment, and can induce significant health benefits [80]. In particular, a long-term (6 months) home based supervised resistance training program that focused on upper and lower limb exercises including leg abduction, sitting, standing from a chair and calf exercises (4 times per week; 15 repetitions; 4 sets per session), showed improvements in lower limb strength in older individuals [7]. In addition, unsupervised home-based resistance training induced improvements in QoL, lower limb muscle strength and reduced postural sway in community-dwelling older adults [81]. Therefore, performing strength-resistance training results in improvements in physical functioning and wellness. However, more research on the effect of strength-resistance training carried out at home for the older population are needed to better understand the effects of frequency (3 or more days per week), intensity (from very light (40% 1RM) to vigorous (70–84% 1RM)), repetition number (from <9 to >18), number of sets (3 or more) and the type of exercises as single or multijoint (Table 5 and Table 6).

### 3.4. Chronic Diseases

Physical inactivity, among older adults, represents one of the highest risk factors for mortality worldwide and a major contributor to chronic disease [76]. It is important to emphasize that during COVID-19 pandemic outbreak, there was a significant decrease in PA levels [82], particularly among older adults. For this reason, new and effective strategies to enhance PA levels for older individuals who are living with chronic diseases should be considered.

*Pulmonary diseases*. With the technological progress in virtual reality, new and innovative approaches to improve traditional physical therapy and rehabilitation programs during quarantine are needed [83]. It has been shown that the use of virtual reality in rehabilitation, such as exergames, improves patient engagement in therapy. In particular, patients show increased determination and motivation to train [83]. In a recent study [84], a console based system (model Xbox 360) was used to train 68 patients with moderate to severe COPD (initial physical capacity: 6.3 s (timed up and go test)) in a 2-week exergame program. The results showed improvements in physical fitness, particularly in postural control, strength and mobility. Similarly, an 8-week home-based program using a virtual reality headset showed improvements in respiratory function, physical function (increased strength, mobility, and flexibility) and QoL in COPD older adults [85]. Furthermore, pulmonary rehabilitation, including cardiovascular training as high intensity interval training (HIIT), improved physical fitness and respiratory function in patients with COPD [86]. Virtual reality HIIT training with subjects wearing headset connected to gaming computers and instructed to pedal on a bike showed improvements in respiratory function and social experiences in community-dwelling older adults with COPD [86].

*Fractures*. Supervised multicomponent home-based balance and strength training are often used to improve QoL and reduce risk of falls in older adults [87]. In fact, semi-supervised (one visit twice a week) home-based rehabilitation programs, including strengthening exercises such as sit-to-stand, forward and lateral step-ups onto a small block, semi squats and heel raises in standing (2 times per week; 3 months) and balance exercises such as standing with a decreased base of support, forwards and sideways stepping/walking, and graded reaching activities in standing, improved QoL and decreased the rate of falls in older persons recovering after hip fracture [88]. Supervised multicomponent home-based programs including weight bearing, gait training, strength-resistance, aerobic and functional exercises (2 times per week for 60 min; 9 weeks) had beneficial effects especially in mobility and walking speed in older patients after hip fracture surgery [88]. In addition, a short-term (4 months) home-based program regarding walking training, lower limb muscle strengthening, balance exercises and assisted ambulation (three times per week in the first two months and twice a week for the following two months) improved physical function in general in older adults after hip fracture [89].

*Sarcopenia*. When the health status of older adults during the COVID-19 outbreak, characterized by sedentary behaviors, is taken into account, it puts older adults at higher risk of sarcopenia [90]. Regular levels of PA have been associated with reducing the detrimental effects of sarcopenia. Structured strength-resistance PA programs were shown to be effective in sarcopenia prevention and in muscle mass function among older adults, compared to aerobic programs [13]. In particular, long-term (6 months) home-based resistance programs showed improvements in lower limb muscle strength among older adults [7]. However, few studies have investigated the effects of the pandemic on sarcopenia in older adults. The development of effective strategies including exercises carried-out in a safe home environment, such as sitting and getting up from a chair, holding a chair, and going up and down steps, are recommended.

*Type 2 diabetes*. Multicomponent (endurance and strength training) home-based programs, along with increased regular PA levels, showed beneficial effects on insulin sensitivity and glycemic control [91]. Aerobic and strength-resistance home-based programs (2–3 times per week) focusing on warm-up, upper and lower limbs strength exercises and cool-down, improved physical fitness in older people with type 2 diabetes [91]. Similarly, long-term (16 weeks) strength-resistance home-based program including chest press, shoulder press, overhead pull-down, lateral shoulder raise, biceps curl, triceps extension, hip flexion, hip extension, calf raise, legs extension, squats and seated rows (8–10 repetitions) led to significant improvements in glucose homeostasis and systolic blood pressure of the older adults [92]. Therefore, for older adults living with chronic conditions, the promotion and the implementation of home-based exercise programs during the COVID-19 outbreak to increase PA levels and counteract the detrimental effects of diseases is fundamental.

*Osteoarticular disorders*. In a time of restrictions like the COVID-19 pandemic, the goal is to provide exercise recommendations for frail older adults [93]. Combined PA programs including aerobic, strength-resistance, proprioceptive and mobility exercises are recommended by clinical guidelines [94] in the management of osteoarticular disorders. In particular, evidence [93] encourages the older population with osteoarticular disorders to walk for at least 10 min per day. Nevertheless, no studies have investigated different exercise frequency, intensity, volume and progression of home-based PA programs during COVID-19 (Table 7 and Table 8).

## 4. Discussion

The purpose of this narrative review was to summarize the literature on the effects of home-based PA, specifically focusing on postural control, fall prevention, mobility, strength-resistance training and QoL, before and during the COVID-19 pandemic, in older adults.

In general, home-based PA programs have been used among subjects living with physical or neurological deficits, such as hospitalized or long-term care residents [14], Parkinson’s disease [95] or disability [96]. However, in recent years, home-based programs have received increasing attention in elderly people in general. Several home-based PA programs have been proposed. Major beneficial effects have been observed following multicomponent home-based programs including low-intensity balance and muscle-strengthening exercises. In particular, supervised and semi-supervised programs showed improvements in mobility [37,38] and strength [7,44,45,46]. Results suggest that unsupervised programs were less effective than supervised programs [47], probably because exercises were based on recommendations developed by an expert with no individual supervision during the sessions. Therefore, the introduction of illustrated exercise books or on-line video exercise classes to highlight the correct movement execution is recommended. The consequences of sedentary behaviors are consistently found to be an increased risk and a potential worsening of the chronic health conditions. For adults living with chronic health conditions such as pulmonary disease, fractures, sarcopenia, type 2 diabetes and osteoarticular disorders, PA represents a protective factor for the management and prevention of these conditions [4]. However, the beneficial effects of PA are different for each disease. In particular, home-based PA programs including aerobic activities improved specific markers, including QoL in subjects with type 2 diabetes [60] and osteoarticular disorders [61]. Muscle-strengthening programs reduced aspiration pneumonia in people with pulmonary disease [21] and improved the QoL in type 2 diabetes [60]. Home-based PA programs focused on balance activities had beneficial effects in pulmonary disease [48] and fractures [52,53], and led to a decreased risk of falls. A higher beneficial effect of PA has been observed following a multicomponent PA program (balance training and muscle strengthening) representing a valuable preventive strategy for falls and possible consequent bone fractures in elderly [52,53]. Achieving the recommended levels of PA [4] led to significant improvements in physical conditions, balance, muscular functional/physical performance, and a positive influence on the QoL in the older adults with sarcopenia [56,57]. Thus, home-based PA programs are safe and cost-effective and play a key role in the prevention and managing of chronic diseases in older living with chronic diseases.

Considering the COVID-19 pandemic, beyond the risk of acute illness, hospitalization and mortality, the major consequence for the whole population, especially older adults, has been the inability to perform regular activities of daily life and maintain regular levels of PA. The combined effect of changes in lifestyle behavior, home confinement and the aging process have a significant negative effect on physical well-being [97]. The reduction in mobility and muscle strength and the loss of aerobic fitness are the major result of this process [76]. An appropriate strategy to stay physically active and reduce social isolation is represented by home-based programs [17]. In fact, during quarantine there was a proliferation of online classes with or without the supervision of experts to train and stay/become physical active, easily accessible with technological devices such as tablets, computers and phones. Supervised, with respect to unsupervised, multicomponent home-based programs based on strength-resistance and functional exercises reduce the risk of falls in older adults [72]. Other beneficial effects were observed consequent to performing long-term and multicomponent programs on mobility, balance and strength in reducing mobility decline [79] and improving QoL [81]. Additionally, exergames, a new and enjoyable training option, have been used to decrease social isolation, improve postural control and encourage PA levels among older people [73]. In recent years, the development of the video game market has strengthened the offer of exergames designed to be used as easier, enjoyable and valuable training tools compared to the traditional exercise programs [98,99,100]. Combined elements of health, games and fitness, and the possibility to connect individuals with specialists via on-line connection [101], could have the potential to improve QoL and physical functions. Furthermore, the possibility of providing specific exergames applications for computers or phones might have a positive effect on social distancing. In this regard, the implementation of functions of online platforms (such as Google Meet, Zoom or Webex Meet) to promote the use of exergames planned for older adults would allow individuals to play or compete with each other in a safe home environment. Therefore, exergames could elicit improvements in overall aspects of health, and promote the opportunity to stay active and socially healthy in apparently healthy or older adults with physical/mental limitations. Another critical consequence of physical inactivity induced by COVID-19 home-confinement is the increased risk of the onset of new chronic health conditions. In this context, especially in rehabilitation setting, virtual PA has been proposed. Regarding rehabilitation programs for pulmonary disease, virtual PA showed improvements in physical fitness [84], physical function and QoL [85] in older adults with COPD. A major beneficial effect is observed when older adults performed multicomponent (balance, strength-resistance and functional exercises) home-based exercise which resulted in improved QoL, reduced rate of falls [87], mobility and walking speed in older after fractures [88]. Aerobic and strength-resistance home-based programs [91,92] had beneficial effects on insulin sensitivity and glycemic control in type 2 diabetes. Lastly, structured strength-resistance and aerobic PA programs seem to be effective in reducing the detrimental effects of sarcopenia and osteoarticular disorders during the pandemic. However, further studies focused on the effects of the pandemic on these diseases in older adults are recommended.

Therefore, the development of mobile applications, specifically designed for older adults, has provided the opportunity to exercise using on-line devices during the COVID-19 pandemic while maintaining social distancing but minimizing social isolation. However, the major limit that emerged and has caused dropout in virtual PA programs was due to connectivity problems or the inability to use on-line devices [102]. For this reason, the implementation of on-line device education, to encourage and promote home-based programs or virtual reality, especially for older adults, is needed. 

Lastly, regular involvement of apparently healthy older adults or those with specific chronic diseases in home-based programs before and during COVID-19 to improve the normal levels of PA represent a potential strategy to improve QoL and to increase physical well-being.

This narrative review can help the general aging population to carry out PA in a safe home environment when physical inability or confinement are required. Several limitations need to be acknowledged for the present narrative review. The reviewed sample was limited to older adults. It would be necessary to broaden the search to other populations such as children, adolescents, adults, athletes and/or individuals with neurological diseases. In addition, no systematic review was carried out, and the exclusion of some studies may have affected the entirety of the data presented.

## 5. Conclusions

PA can preserve physical function in general, maintaining individual autonomy for a longer period and delaying the onset of chronic diseases. Before and during COVID-19 home-confinement, home-based PA programs represent an alternative and effective method for apparently healthy older adults, or those with specific chronic diseases, to stay physically active. The use of technological devices such as tablets, smartphones, and exergames is useful for engaging in PA at home at any time of the day, leading to an increased compliance of the older adults to PA programs. Beneficial effects produced by PA are specific for each disease, and it is possible to structure a specific exercise program in order to improve physical conditions and slow down the physiological aging process. Combined balance and strength training is particularly useful in preventing falls and their consequences, such as fractures, mobility limitations, resistance and strength. In addition, the use of inexpensive and feasible online devices seems to be a potential strategy to be used to stay physically active.

In conclusion, home-based programs induce improvements in physical functions and QoL in older people with or without co-morbidities, and they should be strongly considered, in the near future, as one of the ways to increase physical well-being among older adults.

## Figures and Tables

**Table 1:** The principles of exercise prescription before COVID-19.

References	Duration; Frequency	Intensity	Time	Type	Volume	Progression
[3]	6 to 24 months; 1 to 3 d x wk	NA	2 h	Balance	NA	NA
[16]	8 wk; 3 d x wk	NA	NA	Balance	NA	NA
[28]	8 wk; 3 d x wk	NA	NA	Balance	NA	NA
[29]	16 wk; 4 d x wk	NA	20 to 30 min	Balance	NA	NA
[30]	12 months; 3 d x wk	NA	40 min	Balance	NA	NA
[31]	4 wk; 3 d x wk	NA	60 min	Balance	NA	NA
[32]	12 wk; 3 d x wk	Moderate to high	60 min	Balance	NA	NA
[37]	8 wk; 2 to 3 d x wk	Moderate to high	40 min	Mobility	NA	NA
[38]	12 wk; 3 d x wk	RPE 13/15 Borg 6-20	40 min	Mobility	NA	NA
[39]	6 months; 3 d x wk	NA	NA	Mobility	NA	Resistance bands
[44]	6 months; 3 d x wk	RPE 7/8 CR 10	NA	Strength-resistance	8 rep	NA
[45]	12 wk; 3 d x wk	NA	NA	Strength-resistance	10–15 rep	Resistance bands
[46]	12 months	NA	NA	Strength-resistance	NA	NA
[47]	12 wk; 3 d x wk	RPE 12/16 Borg 6-20	NA	Strength-resistance	8–15 rep	NA

NA: not applicable; d: day; x: the number of training days per week; wk: week; min: minute; h: hour; rep: repetitions; RPE: rate of perceived exertion; Borg: Borg scale from 6 to 20; CR 10: category-ratio at number 10.

**Table 2:** The main outcomes before COVID-19.

References	Outcomes (Δ)	Pre	Post	Diff. Pre-Post
[16]	Cognitive status	24.25	24.56	0.31 (AU)
	ADL	87.81	88.88	1.07 (AU)
	PHS	7.8	8.13	0.33 (AU)
	SEE	50.06	65.69	15.63 (AU)
	OEE	49.25	47.69	−1.56 (AU)
[28]	TUG	15.04	12.1	−2.94 (s)
	BBS	51	55	4 (AU)
	One-leg stance	8.3	20.8	12.5 (s)
	Tandem standing	21.2	35	13.8 (s)
[30]	Figure-of-8 running	20.6	19.4	−1.2 (s)
[31]	Dynamic balance	7.2	6.1	−1.1 (s)
[32]	Low grip strength	15.71	18.84	3.13 (kg)
	TUG	12.21	10.33	−1.88 (s)
	BBS	49.12	52.68	3.56 (AU)
	VO_2max_	26.53	27.59	1.06 (mL/kg/min)
[33]	Balance	10.91	13.41	2.5 (s)
	Gait speed	6.25	8.25	2 (s)
[37]	6MWT	318.8	404.8	86 (m)
	15-m walking test	15.9	12.7	−3.2 (s)
[44]	Tandem walk	47.9	30.9	−17 (s)
	One-legged stance	8.5	15.7	7.2 (s)
	Maximal gait	1.7	1.5	−0.2 (m/s)
	6MWT	1227	1324	97 (ft)
	Hand grip	21.8	22.4	0.6 (kg/f)
	Knee extension 1RM	17.1	20.2	3.1 (kg)
[45]	TUG	7.23	7.16	−0.07 (s)
	4-m walk test	0.98	1.04	0.06 (m/s)
	5-times sit to stand	11.68	10.58	−1.1 (s)
	4-step stair climb	2.73	3.16	0.43 (s)
	SPPB	11	11.3	0.3 (AU)
[46]	MWS	102	111	−2.2 (m/min)
	TUG	7.2	7	−0.2 (s)
	RSTL	26.9	25.9	−1 (s)
	LSTL	25.1	25.1	0 (s)
[47]	ROM	102.7	100.5	−2.2 (mm/s)
	Stride velocity	1.38	1.46	0.08 (m/s)
	TUG	9.91	8.78	−1.13 (s)

Diff.: difference; ADL: activities of daily living; PHS: perceived health status; SEE: self-efficacy for exercise; OEE: outcome expectations for exercise; TUG: timed up and go; BBS: berg balance scale; 6MWT: 6-min walking test; 1RM: one repetition maximus; SPPB: short physical performance battery; MWS: maximum walking speed; RSTL: standing time on the right leg; LSTL: standing time on the left leg; ROM: range of motion; AU: arbitrary units; s: second; kg: kilogram; mL: milliliter; min: minute; ft: feet; mm: millisecond; m: meter.

**Table 3:** The principles of exercise prescription of chronic diseases before COVID-19.

References	Duration; Frequency	Intensity	Time	Type	Volume	Progression
[48]	6 months; 3 d x wk	NA	NA	Stretching; balance; breathing; dual-tasks training	NA	NA
[49]	Daily	Low-intensity (30–40% Plmax)	15 min	Sitting calisthenics; respiratory stretching; walking	NA	NA
[50]	7 wk; 6 d x wk	30% Plmax	NA	Inspiratory training	NA	Increase 5% Plmax
[52]	6 months; 3 d x wk	NA	NA	Function-oriented exercises	NA	NA
[53]	6 to 24 months; 2 d x wk	NA	NA	Balance; strength	8–10 rep; 10–15 rep	NA
[56]	9 wk; 3 d x wk	NA	NA	Strength-resistance	NA	NA
[57]	3 months; 2 d x wk	NA	NA	Strength-resistance; balance	12 rep	NA
[59]	NA	High-intensity (50–80% 1RM)	NA	Strength-resistance	NA	NA
[60]	6 months; 2 d x wk	75% 1RM	40 s	Strength; aerobic	NA	Increase 20% 1RM
[61]	6 d x wk	Low-intensity	10 min	Intermittent walking	NA	NA

NA: not applicable; d: day; x: the number of training days per week; wk: week; min: minute; s: second; rep: repetitions; Plmax: maximum inspiratory pressure; 1RM: one repetition maximum.

**Table 4:** The main outcomes of chronic diseases before COVID-19.

References	Outcomes (Δ)	Pre	Post	Diff. Pre-Post
[48]	5-min walking time	2.96	2.78	−0.18 (s)
	TUG	6.83	6.6	−0.23 (s)
	Sit and reach test	14.9	17.7	2.8 (cm)
[49]	6MWD	404	467	63 (m)
[53]	SPPB	5.9	6.3	0.4 (AU)
	BBS	40.8	45.6	4.8 (AU)
	Leg strength	25.5	29.9	4.4 (AU)
[56]	TUG	6.16	5.63	−053 (s)
	5x chair rise	10.95	9.54	−1.41 (s)
	SPPB	11.38	11.88	0.5 (AU)
	10m test habitual	1.38	1.43	0.05 (m/s)
	10m test fast	1.9	2.01	0.11 (m/s)
[57]	TUG	9.67	8.82	−0.85 (s)
	4m test	4.47	3.71	−0.76 (s)
	Gait speed	0.9	1.11	0.21 (m/s^2^)
	Chair stand test	12.29	11.41	−0.88 (s)
	Handgrip strength	19.2	18.68	0.52 (kg)

Diff.: difference; TUG: timed up and go; 6MWD: 6-min walking distance; SPPB: short physical performance battery; BBS: berg balance scale; s: second; cm: centimeter; AU: arbitrary units; m: meter; kg: kilogram; x: the number of training days per week.

**Table 5:** The principles of exercise prescription during COVID-19.

References	Duration; Frequency	Intensity	Time	Type	Volume	Progression
[72]	20 sessions; 2 d x wk	NA	1 h	Proprioceptive	NA	NA
[73]	12 wk; 1 d x wk	NA	NA	Exergames	NA	NA
[78]	7 wk; 5 d x wk	NA	20 min	Mobility	NA	NA
[79]	8 wk; 3 d x wk	NA	40 min	Strength; balance; mobility	NA	NA
[7]	6 months; 4 d x wk	NA	NA	Strength	15 rep	Increase sets/rep

NA: not applicable; wk: week; d: day; x: the number of training days per week; min: minute; h: hour; rep: repetitions.

**Table 6:** The main outcomes during COVID-19.

References	Outcomes (Δ)	Pre	Post	Diff. Pre-Post
[7]	Chair-stand-test	14	16	2 (rep)
	Handgrip strength	30.04	28.2	−2.2 (kg)
	Mini-BEST test	25	26	1 (AU)
	Thigh extensors strength	333	329	−4 (n)
	Thigh flexors strength	176	178	2 (n)
[78]	KSS knee score	49.7	89.5	39.8 (AU)
	KSS function score	37.02	91.8	54.6 (AU)
	ROM	88.2	116.7	28.5 (°)
	VAS	5.5	0.8	−4.7 (AU)
[79]	Tandem stance	68.1	96.4	28.3 (s)
	TUG	44.4	35.5	−8.9 (s)

Diff.: difference; KSS: Knee Society Score; ROM: range of motion; VAS: visual analogue scale; TUG: timed up and go; rep: repetitions; kg: kilogram; AU: arbitrary units; n: Newton; °: degree; s: second.

**Table 7:** The principles of exercise prescription of chronic diseases during COVID-19.

References	Duration; Frequency	Intensity	Time	Type	Volume	Progression
[84]	2 wk	NA	NA	Exergames	NA	NA
[85]	8 wk	NA	NA	Virtual realty	NA	NA
[86]	8 wk	NA	NA	HIIT	NA	NA
[88]	12 wk; 2 d x wk	NA	NA	Strength; balance	NA	NA
[89]	4 months; 3 d x wk	NA	NA	Walking; strength; balance	NA	NA
[92]	16 wk	NA	NA	Strength-resistance	8–10 rep	NA
[7]	6 months	NA	NA	Resistance	NA	NA
[93]	Daily	NA	10 min	Walking	NA	NA

NA: not applicable; wk: week; d: day; x: the number of training days per week; min: minute; HIIT: high intensity interval training; rep: repetitions.

**Table 8:** The main outcomes of chronic diseases during COVID-19.

References	Outcomes (Δ)	Pre	Post	Diff. Pre-Post
[7]	Chair-stand-test	14	16	2 (rep)
	Handgrip strength	30.04	28.2	−2.2 (kg)
	Mini-BEST test	25	26	1 (AU)
	Thigh extensors strength	333	329	−4 (n)
	Thigh flexors strength	176	178	2 (n)
[84]	Arm curl	18.6	21.8	3.2 (rep)
	Chair stand	14.3	16.6	2.3 (rep)
	Back scratch	−6.1	4	2.1 (cm)
	Sit and reach	0.7	3.4	2.7 (cm)
	Up and go	6	5.3	−0.7 (s)
	6MWT	469.9	508.4	38.5 (m)
[85]	SPPB	6.78	8.43	1.65 (AU)
	CRQ-dyspnea	2.22	2.96	0.74 (AU)
	CRQ-fatigue	3.11	3.27	0.16 (AU)
	CRQ-emotional	3.85	4.36	0.51 (AU)
	CRQ-mastery	3.83	4.22	0.39 (AU)
[89]	Barthel Index	54.17	82.5	28.33 (AU)
	FAC	0	3.33	3.33 (AU)
	Hip flexion	28.33	107.5	79.17 (°)
	Hip abduction	11.67	35.83	24.16 (°)

Diff.: difference; 6MWT: 6-min walking test; SPPB: short physical performance battery; CRQ: chronic respiratory questionnaire; FAC: Functional Ambulation Category; rep: repetition; kg: kilogram; AU: arbitrary units; n: Newton; cm: centimeter; s: second; m: meter; °: degree.

## Data Availability

The papers included in the present narrative review are available on reasonable request from the corresponding author.
